# Effect of H_2_S on the circadian rhythm of mouse hepatocytes

**DOI:** 10.1186/1476-511X-11-23

**Published:** 2012-02-08

**Authors:** Zhanxian Shang, Chao Lu, Sifeng Chen, Luchun Hua, Ruizhe Qian

**Affiliations:** 1 Department of Physiology and Pathophysiology, Fudan University Shanghai Medical College, Shanghai 200032, PR China; 2 Department of Surgery, Huashan Hospital affiliated to Fudan University, Shanghai 200040, PR China

**Keywords:** Hydrogen sulfide, sirt1, circadian clock genes, metabolism-related genes, lipid

## Abstract

**Background:**

Dysregulation of circadian rhythms can contribute to diseases of lipid metabolism. NAD-dependent deacetylase sirtuin-1(SIRT1) is an important hub which links lipid metabolism with circadian clock by its deacetylation activity depends on intracellular NAD^+^/NADH content ratio. Hydrogen sulfide (H_2_S) is an endogenous reductant which can affect the intracellular redox state. Therefore, we hypothesized that exogenous H_2_S can affect the expression of circadian clock genes mediated by sirt1 thereby affecting body's lipid metabolism. And also because the liver is a typical peripheral circadian clock oscillator that is intimately linked to lipid metabolism. Thus the effect of H_2_S were observed on 24-hour dynamic expression of 4 central circadian clock genes and sirt1gene in primary cultured hepatocytes.

**Results:**

We established a hepatocyte model that showed a circadian rhythm by serum shock method. And detected that the expression level and the peak of circadian clock genes decreased gradually and H_2_S could maintain the expression and amplitude of circadian clock genes such as Clock, Per2, Bmal1 and Rev-erbαwithin a certain period time. Accordingly the expression level of sirt1 in H_2_S group was significantly higher than that in the control group.

**Conclusion:**

Exogenous reductant H_2_S maintain the circadian rhythm of clock gene in isolated liver cells. We speculated that H_2_S has changed NAD^+^/NADH content ratio in hepatocytes and enhanced the activity of SIRT1 protein directly or indirectly, so as to maintain the rhythm of expression of circadian clock genes, they play a role in the prevention and treatment of lipid metabolism-related disease caused by the biological clock disorders.

## Background

To adapt the changes of environment, all species on the earth have a life cycle synchronized approximately with the circadian rhythm of the planet. In mammals, the central circadian clock is located in hypothalamic supraoptic nucleus (SCN) [1] with a sensitivity of the outside light signal. Peripheral circadian clock is regulated by the central circadian clock. The negative feedback loop composed of circadian clock genes and their expression products oscillate autonomously. Circadian rhythm of life on earth is realized in this way [2]. Main circadian clock genes includes Clock, Bmal1, Per2, Rev-erbα, Cry1 etc. [3,4] Disorder of circadian rhythms can contribute to disease. Conversely, metabolic signals also feed back into the circadian system, modulating circadian gene expression and behavior [5]. Many peripheral tissues have a rhythmic expression of circadian genes as well as SCN. Especially products of circadian genes in fat, liver, heart, pancreatic β-cell are all directly or indirectly involved in energy metabolism [6,7]. Emerging evidence suggests that there are food-induced oscillators independent of the SCN [8-10]. The feedback loop formed by circadian clock genes and their products regulate the expression of different clock controlled genes, and then regulate the physiological, behavioral activities of the body [11]. Between the central and peripheral organs and between tissues of different peripheral organs, expression of clock controlled genes have significant specificity [12]. The liver is critical organ for lipid metabolism. It is very sensitive to changes in internal environment. Expression of many circadian genes and lipid metabolism-related genes shows a clear rhythm in hepatocytes. That is why we choose primary hepatocytes for our experiment.

Sirtuin-1 (NAD-dependent deacetylase sirtuin-1, SIRT1) is an NAD^+^-dependent deacetylase encoded by the sirt1gene. It is a key regulator of metabolic homeostasis [13] and can enhance gluconeogenesis and lipolysis, regulate differentiation of adipocyte, promote insulin secretion, and enhance tissue sensitivity to insulin [14]. In addition, SIRT1 exerts vasculoprotective effects by anti-inflammation, anti-apoptosis, blood vessel relaxation, inhibition of foam cell formation and etc [15]. Appealingly, SIRT1 is an integral part of the circadain clock operation. SIRT1 enhances the activation of ROR (RAR-related orphan receptor) on the transcription of mBmal1 by activating PGC-1α (peroxisome proliferator-activated receptor-γ coactivator-1α) [16,17]. CLOCK-BMAL1 heterodimer is the core components of circadian clock. It binds to the upstream E-BOX to regulate the expression of other clock genes. However, the acetylated dimer is not active. SIRT1 regulates the function of CLOCK-BMAL1 heterodimer through deacetylation to mediate energy metabolism and circadian clock [17].

Deacetylation of SIRT1 depends on the NAD^+^/NADH content ratio in cytoplasm. While H_2_S is endogenous reductant and affects the intracellular redox state. H_2_S is one of degradation products of endogenous sulfur amino acid. H_2_S plays a vasculoprotective role in mammalian atherosclerosis. Firstly, H_2_S can inhibit the proliferation of vascular smooth muscle and the latter is considered to be an important part of the formation of atherosclerosis [18]. Secondly, metabolism of sulfur amino acids is closely related to lipid metabolism. Some patients with coronary artery disease have no traditional risk factors such as hypertension, hyperlipidemia, diabetes, smoking, etc. Hyperhomocysteinaemia induced by deposition of high homocysteine is now considered one of the independent risk factors of atherosclerosis [19]. Thirdly, H2S can inhibit the oxidative modification of low-density lipoprotein (LDL) in vitro [20]. In addition, of H2S restore vasodilation and inhibit angiogenesis via vasodilation [21,22]. H2S through these pathways involved in lipid metabolism-related diseases.

Then we hypothesized that H_2_S affect circadian clock genes expression by changing the activity of SIRT1 and thereby affecting the cellular lipid metabolism. So through the serum shock method we tried to establish a model of primary hepatocytes which has rhythmic expression of circadian clock genes and then detected the effects of H_2_S on the gene expression.

## Results

### Primary cultured hepatocytes

**Figure 1:**
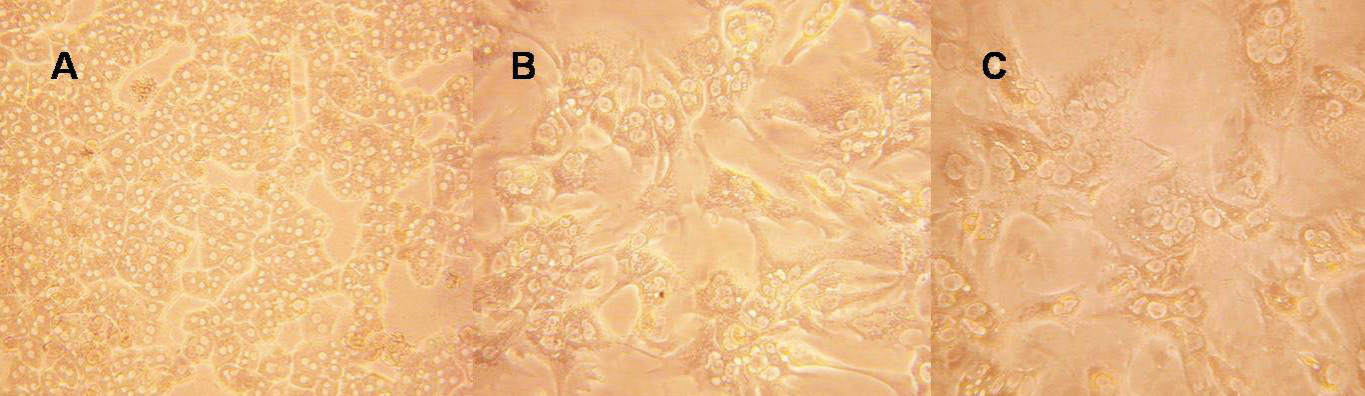
**Primary hepatocytes before and after serum shock**: **A **After cultured for 4 hours, the hepatocytes adhered, and formed into hepatic cord; B After cultured by medium without serum for 24 hours, hepatocytes extend pseudopodia; C After cultured by medium without serum for 4 days or after serum shock for 3 days, cell-fusion and fibroblastization could be observed.(A × 200;B × 400;C × 400).

Newly isolated hepatocytes of mice were spherical with two striking nucleus. After cultured for 4 hours the hepatocytes adhered and formed into hepatic cord (Figure 1A). Hepatocytes were polygonal, tightly packed with clear nucleolus. After cultured by medium without serum for 24 hours, hepatocytes extend pseudopodium (Figure 1B). Cell condition has been restored to some extent after serum shock. After cultured by medium without serum for 4 days or after serum shock for 3 days, cell-fusion and fibroblastization could be observed. Cell condition decreased significantly (Figure 1C). Proliferation of hepatocytes was not observed in the culture process.

### Diurnal expression patterns of circadian genes in control group

**Figure 2:**
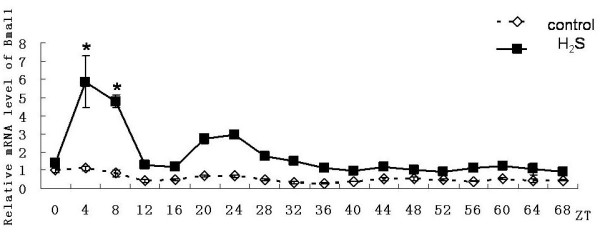
**Effect on expression of Bmal1 gene in primary cultured hepatocytes**. Horizontal axis shows the period and vertical axis shows the amplitude. The mRNA levels of Bmal1 gene were normalized to GAPDH mRNA. Each value represents the mean ± SD (n = 3). The expression differences were assessed by one-way ANOVA.

**Figure 3:**
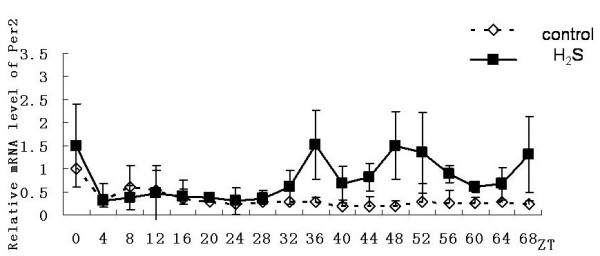
**Effect on expression of Per2 gene in primary cultured hepatocytes**. Horizontal axis shows the period and vertical axis shows the amplitude. The mRNA levels of Per2 gene were normalized to GAPDH mRNA. Each value represents the mean ± SD (n = 3). The expression differences were assessed by one-way ANOVA.

**Figure 4:**
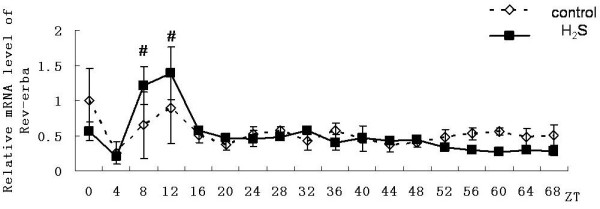
**Effect on expression of Rev-erbα gene in primary cultured hepatocytes**. Horizontal axis shows the period and vertical axis shows the amplitude. The mRNA levels of Rev-erbα gene were normalized to GAPDH mRNA. Each value represents the mean ± SD (n = 3). The expression differences were assessed by one-way ANOVA.

In the control group, genes of mBmal1, mPer2 and mRev-erbαhad shown a 24-hour cyclical rhythm. Expression of Bmal1gene from the ZT0 point (According Zeitgaber Time, the time adding 50% horse serum was denoted as ZT0) showed a rhythmicity. and the first expression peak occured at ZT8, the second peak occured at ZT32, the last peak occured at ZT56. Peaks decreased gradually and fluctuating rhythm was no longer obvious after ZT64(Figure 2); Expression of Per2 showed a rhythmicity from ZT4 point, and the first expression peak occured at ZT8, the second peak occurred at ZT32, the last peak occured at ZT56. Rhythm can be maintained more than three days(Figure 3); Expression of Rev-erbαgene showed a rhythmicity form ZT8 point and lasted three days. The first expression peak occured at ZT12, and the second peak occured at ZT36, the last peak occured at ZT60. Peaks decreased gradually (Figure 4).

### Diurnal expression patterns of circadian clock genes in H_2_S group

**Figure 5:**
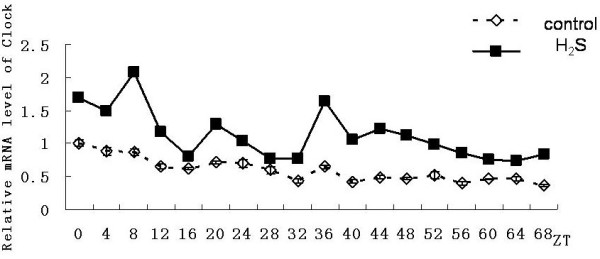
**Effect on expression of Clock gene in primary cultured hepatocytes**. Horizontal axis shows the period and vertical axis shows the amplitude. The mRNA levels of Clock gene were normalized to GAPDH mRNA. Each value represents the mean ± SD (n = 3). The expression differences were assessed by one-way ANOVA.

In the hydrogen sulfide group, same with the control group genes of Bmal1, Per2 and Rev-erbαhad also shown a 24-hour cyclical rhythm. Although still no significant rhythmicity was shown in the expression of Clock gene, the expression level Clock gene was significantly higher than in control group (n = 3, p < 0.05). However it also reduced gradually (Figure 5), the expression level of Per2 gene was significantly higher than that in control group from the ZT36 point (n = 3, p < 0.05). Until to the ZT40 point it showed rhythmicity and the amplitude was significantly higher than control group (Figure 3). The emergence time of rhythm and peak of the Bmal1gene and Rev-erbαgene expression were both synchronized with the control group. And peaks of the genes both reduced gradually. For mBmal1gene, not only the expression level but also the volatility were significantly increased (n = 3,*p < 0.01). However the maintain time was not more than 48 hours (Figure 2). At ZT8 and ZT12 time point the expression level of Rev-erbαgene was significantly higher than that in control group(n = 3,#p < 0.05). However subsequently the amplitude decreased rapidly and the expression level decreased under the control group from the third day (Figure 4).

### Diurnal expression patterns of sirt1gene

**Figure 6:**
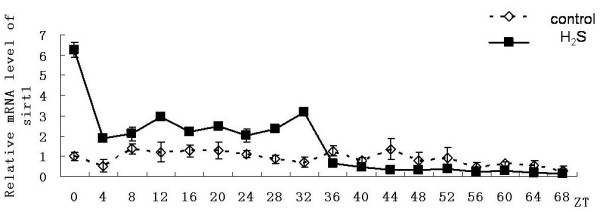
**Effect on expression of sirt1 gene in primary cultured hepatocytes**. Horizontal axis shows the period and vertical axis shows the amplitude. The mRNA levels of sirt1 gene were normalized to GAPDH mRNA. Each value represents the mean ± SD (n = 3). The expression differences were assessed by one-way ANOVA.

In addition to circadian clock genes' expression, we also detected the diurnal expression patterns of sirt1 gene. The results showed that in the control group there was a small peak of expression of sirt1 gene at ZT8 point. And then, gradually, the expression level decreased. No significant circadian rhythm was seen throughout the experiment. While in the H_2_S group, in the first 32 hours after serum shock, the expression level of sirt1gene was significantly higher than that in the control group (Figure 6).

## Discussion

Oscillators independent of the SCN exist in peripheral tissues [10]. They regulate various physiological and biochemical activities specifically. Such as mouse embryonic fibroblasts (MEFs) show circadian rhythm after serum shock [23]. We also found mouse myocardial cells cultured in vitro in pre-trial were able to maintain the circadian rhythm at 2 ~ 3d. This indicates that expression of circadian clock genes in peripheral tissue generates independent rhythmic vibration. The liver is a key organ about lipid metabolism. It receive signals of energy and hormone fome internal environment, regulate the β-oxidation of fatty acid and lipoprotein uptake and release [24]. The expression of many genes in liver cells shows a significant circadian rhythm. In our study, H_2_S in the form of NaHS [25] was prepared prior to use temporarily. Two-thirds of the ions exist in the form of hydrogen sulfide in the NaHS solution, one-third of the sulfur in the form of hydrogen ions. As the H2S is a gas, the stability of H2S in the solution varied depending on the initial H2S concentrations. At the highest concentration tested (1 mM), a drop of the H2S concentration around 15% within 30 min was observed [26]. Of note, at 37°C, the concentration of H2S in the solution is very stable below 1 mM [27,28] and does not affect the PH value of culture medium [29]. So we tested at low dose as much as possible. However, we found that the experimental group showed unstable rhythms at the concentration of 2.5 × 10-4 mol/L, and even showed no rhythm sometimes. Then we increased the dose. However, the concentration of freshly preparad NaHS was far below 1 mM. And determined the final concentration of H2S to use is 5 × 10^-4^mol/L, and the solution is added every 24 hours after the serum shock.

The expression of Clock gene in liver in vivo were constant, therefore we found that in isolated hepatocytes, expression level decreased gradually, but no noticeable circadian changes were observed in 24-hour period(table 1). In the experimental group Clock gene expression was significantly higher than the control group, Clock expression was gradually reduced over time, the two groups expressed almost the same level after ZT48. Accordingly, we concluded that H_2_S can maintain the expression levels of Clock gene in isolated hepatocytes within a certain period of time or H_2_S alleviated the attenuation of Clock gene expression in hepatocytes in vitro.

**Table 1:** Diurnal expression patterns of circadian clock genes and sirt1 gene

Genes	CRc	CRt	RZTc	RZTt	MAc	MAt	MAZTc	MAZTt
Clock	-	-	-	-	-	-	-	-

Bmal1	+	**+**	ZT0	ZT0	1	5.29↑	ZT4	ZT4

Per2	+	**+**	ZT4	ZT44	1	2.53↑	ZT8	ZT48

Rev-erbα	+	**+**	ZT8	ZT8	1	1.56↑	ZT12	ZT12

Sirt1	-	-	-	-	-	-	-	-

Notation: CR, Circadian rhythm; RZT, Appearance of rhythm(ZT); MA, Max Amplitude(relative value); MAZT, Appearance of Max Amplitude(ZT).

Lower case letters in the first line: c:control; t: test

As can be seen from the Table 1, the Bmal1 gene, Per2 gene and Rev-erbα gene have shown a rhythm in the control group. In the H_2_S groups, the expression level and amplitude of three genes were significantly increased. The expression of Bmal1 and Rev-erbα showed a circadian rhythm, peak time of which was synchronized with the control group. However the maintenance of H_2_S on rhythmic expression of Bmal1 gene and Rev-erbα gene did not lasted a longer time than the control group. This showed that H_2_S improved and maintained their volatility of expression transiently. The impaction of H_2_S appeared in a short time and the sensitivity decreased rapidly. We speculated that high expression of Rev-erbα gene at ZT8, ZT12-point activated mCry1 which inhibit the expression of Rev-erbα gene. Rhythmic of Per2 gene expression delayed. So we speculated that H_2_S affect mPer2 gene expression indirectly and promote transcription until more than 36 hours.

In addition, H_2_S did not impact a long-lasting maintenance on the rhythm of the isolated hepatocytes. That might be resulted from the long time for serum-free culture. H_2_S has a protective effect on the maintenance of circadian rhythm of isolated hepatocytes. Then what is the role of the relatively high expression of Sirt1 gene in this process? Our results showed that in the first 32 hours after serum shock, the expression level of sirt1 gene in hydrogen sulfide group were significantly higher than that in the control group. There are two possibilities: (1) H_2_S directly stimulated the expression of sirt1gene by changing the intracellular redox status, thus enhancing the total intracellular biological activity of SIRT1 protein. (2) The change of intracellular ratio of NAD^+^/NADH speed up the activation and inactivation of SIRT1 protein which resulted in the half-life of SIRT1 protein were shortened, and then led to sirt1 gene highly expressed through feedback ways. Finally, a large number of activated SIRT1 protein played a protective role in the rhythmic maintenance of circadian clock genes' expression in isolated hepatocytes. Of course, the mystery revealed awaits further experimental results.

In summary, we established hepatocytes model with circadian rhythm by serum shock method. And found that H_2_S could maintain the expression and the vibration amplitude of Clock, mPer2, mBmal1 and mRev-erbαwithin 48 hours genes. However, there are differences in the time point of occurrence and its duration between different genes. In this process, sirt1 gene sustained high expression. Accordingly, we concluded that exogenous reductant H_2_S has a protective effect on the maintenance of circadian rhythm of circadian clock genes in isolated hepatocytes. The effect is achieved through changing the intracellular ratio of NAD^+^/NADH and enhancing the activity of SIRT1 protein. This conclusion may find a new focus on the prevention and treatment of lipid metabolism related diseases caused by circadian clock disorders.

## Materials and methods

### Cell culture

Selected 8-week-old C57BL/6J mice, and then separated hepatocytes by liver perfusion of collagenase IV(Sigma). After centrifugation, were grown in Williams' Medium E supplemented with 10% FBS(GIBCO), ITS(Sigma)and sodium pyruvate(Gibeco). Four hours after inoculation hepatocytes completely adherent. Cultured for 24 hours with serum free Williams' Medium E medium containing ITS and sodium pyruvate. Subsequently cells were treated with Williams' Medium E containing 50% horse serum for two hours (this process is called serum shock). And then continued to culture with serum free Williams' Medium E medium containing ITS and sodium pyruvate until the end of the experiment. At the same time the experimental group was given NaHS. Every four hours after serum shock cells were harvested. There were 18 time points. All animal experiments were performed according to the criteria of the Medical Laboratory Animal administrative Committee of Shanghai.

### Total RNA extraction and reverse transcription

Total RNA from hepatocytes were isolated with Trizol Reagent (Invitrogen), according to the manufacturer's instructions. 2 μg of total RNA were reversely transcribed and amplified using the ReverTra Ace qPCR RT Kit (TOYOBO).

### Real-time PCR

The real-time PCR was carried out using SYBR Green Real-time PCR Master Mix(Bio-Rad) in a total volume of 20 μl. PCR amplifications were performed in a real-time PCR system (Bio-Rad) in duplicate. The relative quantification of gene expression was analyzed from the measured threshold cycles (CT) by using the 2-ΔΔCt method in the experiment. The data were normalized by determination of the amount of glyceraldehyde-3-phosphate dehydrogenase (GAPDH) mRNA in each sample. In our experience, the target genes names and database names are as follows: Clock[GenBank:NM_007715], Bmal1[GenBank:NM_007489], Per2[GenBank:NM_011066], Rev-erbα[GenBank:NM_145434], Sirt1[GenBank:NM_019812], GAPDH[GenBank:BC_083149]. Primer sequences of the target genes in the present study were found in Genbank as shown in Table 2.

**Table 2:** The primer Sequences Used for PCR Amplification

Gene	**Genebank Accession No**.	Annealing temperature	Primer sequence 5' to 3'
**Clock**	NM_007715	58°C	Forward:CTTCCTGGTAACGCGAGAAAG Reverse:TCGAATCTCACTAGCATCTGACT

**Bmal1**	NM_007489	60°C	Forward:CACTGACTACCAAGAAAGTATG Reverse:ATCCATCTGCTGCCCTGAGA

**Per2**	NM_011066	58°C	Forward:CAGACTCATGATGACAGAGG Reverse:GAGATGTACAGGATCTTCCC

**Rev-erbα**	NM_145434	60°C	Forward:TACATTGGCTCTAGTGGCTCC Reverse:CAGTAGGTGATGGTGGGAAGTA

**Sirt1**	NM_019812	58°C	Forward:GAACAGGTTGCGGGAATC Reverse:AACATGAAGAGGTGTGGGTG

**GAPDH**	BC_083149	60°C	Forward:ACAGCCGCATCTTCTTGTGCAGTA Reverse:GGCCTTGACTGTGCCGTGAATTT

### Statistical analysis

Each value represents the mean ± SD. The values for mRNA levels are presented as relative values in all experiments. The oscillation of each gene expression was evaluated by one-way analysis of variance (ANOVA) by SPSS 16.0 software and the post hoc Student's t-test was used to compare the values between the groups at the same CT point. A probability value < 0.05 was considered statistically significant.

## List of abbreviations

SIRT1: NAD-dependent deacetylase sirtuin-1; Clock: circadian l Locomotor output cycles kaput; Per: Period; Bmal1: brain and muscle Arnt-like protein 1; Rev-erbα: NR1D1 (nuclear receptor subfamily 1, group D, member 1) a member of the nuclear receptor family of intracellular transcription factors; SCN: supraoptic nucleus; ROR: RAR-related orphan receptor; PGC-1α: peroxisome proliferator-activated receptor-γcoactivator-1α; LDL: low-density lipoprotein; ZT: Zeitgeber time(Zeitgeber a German word that literally means 'time giver', it refers to external cues that entrain the endogenous clock); MEFs: mouse embryonic fibroblasts; ITS: Insulin-Transferrin-Selenium-X Supplement; CT: threshold cycles; GAPDH: glyceraldehyde-3-phosphate dehydrogenase.

## Competing interests

The authors declare that they have no competing interests.

## Authors' contributions

ZS carried out all aspects of experiments and data analysis, and drafted the manuscript. CL and SC participated in the design of experiments. LH participated in the design of study and proofread manuscript. RQ conceived of the study and performed the experimental instruction. All authors read and approved the final manuscript.
